# Environment Sound Classification Using a Two-Stream CNN Based on Decision-Level Fusion

**DOI:** 10.3390/s19071733

**Published:** 2019-04-11

**Authors:** Yu Su, Ke Zhang, Jingyu Wang, Kurosh Madani

**Affiliations:** 1 School of Astronautics, Northwestern Polytechnical University (NPU), 127 Youyi Xilu, Xi’an 710072, China; zhangke@nwpu.edu.cn (K.Z.); mesen.wang@gmail.com (J.W.); 2 Signals, Images, and Intelligent Systems Laboratory (LISSI / EA 3956), Université Paris-Est, University Paris-Est Creteil, Senart-FB Institute of Technology, 36-37 rue Charpak, 77127 Lieusaint, France; madani@u-pec.fr

**Keywords:** Auditory Cognition, Environment Sound Classification, Convolutional Neural Network, Dempster—Shafer evidence theory, Fusion Model

## Abstract

With the popularity of using deep learning-based models in various categorization problems and their proven robustness compared to conventional methods, a growing number of researchers have exploited such methods in environment sound classification tasks in recent years. However, the performances of existing models use auditory features like log-mel spectrogram (LM) and mel frequency cepstral coefficient (MFCC), or raw waveform to train deep neural networks for environment sound classification (ESC) are unsatisfactory. In this paper, we first propose two combined features to give a more comprehensive representation of environment sounds Then, a fourfour-layer convolutional neural network (CNN) is presented to improve the performance of ESC with the proposed aggregated features. Finally, the CNN trained with different features are fused using the Dempster–Shafer evidence theory to compose TSCNN-DS model. The experiment results indicate that our combined features with the four-layer CNN are appropriate for environment sound taxonomic problems and dramatically outperform other conventional methods. The proposed TSCNN-DS model achieves a classification accuracy of 97.2%, which is the highest taxonomic accuracy on UrbanSound8K datasets compared to existing models.

## 1. Introduction

Intelligent sound recognition (ISR) is a technology for identifying sound events that exist in the real environment. This method is mainly based on analyzing human auditory awareness characteristics and embedding such percept ability in machines or robots. Environmental sound classification (ESC), also known as sound event recognition, serves as a fundamental and essential step of ISR. The main goal of ESC is to precisely classify the class of a detected sound, such as children playing, car horn and gunshot. With the popular applications of ISR in audio surveillance systems [1] and healthcare [2], the ESC problem has received increasing attention in recent years. Depending on the different properties of various sound sources, sound signals can be roughly classified into human voice, music sound, and environmental sound. Recent developments have brought great improvements in automatic speech recognition (ASR) [3] and music information recognition (MIR) [4]. However, on account of considerably non-stationary characteristics of environmental sounds, this kind of signals cannot be described as speech or music only. We can imagine that the system developed for ASR and MIR will be inefficient when applying to ESC tasks. Therefore, it is essential to develop an efficient ISR system for environment sound recognition.

Environment sound taxonomy generally consists of two basic components: acoustic features and classifiers. In order to extract acoustic features, sound signals are first separated into frames with a cosine window function (Hamming or Hanning window). Then, features are extracted from each frame and this set of features is used as one instance of training or testing [5]. The classification result of one sound is the summation of probabilities predicted for each segment. Features derived from Mel filters: Mel Frequency Cepstral Coefficients (MFCC) and Log-Mel Spectrogram (LM) are two widely used features in ESC [6,7] with acceptable performance, although they are originally developed for ASR. Moreover, a considerable number of research works indicate that combined features performed better than only use one feature set in ESC tasks. While adding more conventional features cannot improve the performance. Hence, a suitable feature aggregate scheme is an essential part of sound taxonomy.

Support-vector machines (SVM), Gaussian mixture model (GMM) extreme learning machine (ELM) are widely used classifiers in sound related classification tasks in the past decades [8,9,10,11] and other categorization problems as well. However, these conventional classifiers are designed to model small variations which result in the lack of time and frequency invariance. In recent years, deep neural network-based models have been proven to be more efficient than traditional classifiers in solving complex categorize problems. The convolutional neural network (CNN) is one of the most commonly used architectures of deep learning models, which could address the former limitations by learning filters that are shifted in both time and frequency [12]. The CNN is designed to process data that come in the form of multiple arrays: 1D for various kinds of sound signals, such as speech and music, and 2D for image or audio spectrograms [13]. Ref. [14] first use the CNN in image recognition and outperform all the traditional methods in the ImageNet Large Scale Visual Recognition Challenge (ILSVRC). CNN has been successfully used for ASR [15] and MIR [4], and this deep architecture is also shown to be extremely powerful in ESC tasks. In 2018 Detection and Classification of Acoustic Scenes and Events challenge (DCASE), 55 of the 97 submissions being based on a CNN architecture [16]. Although great improvement has been achieved by using CNN in ESC problems, however, there is still a long way to go when compared with CNN based image classification algorithms. Therefore, some researchers consider to merge two CNN or fuse CNN with other deep learning models to elevate the performance.

Despite the fact that CNN can solve the limitations of conventional classifiers, the longer temporal context information still cannot be captured by this method. Hence, several works propose to use merged neural networks to address the above-mentioned shortcomings through integrating information from the earlier steps [17,18,19,20]. In these methods, one or more CNNs are used to extract the spatial information with different acoustic features firstly. Then, the outputs are merged by concatenation and feed to recurrent neural network (RNN) layers or another CNN layers for temporal information extraction. Inspired by sensor fusion framework, several research works apply decision-level fusion in ESC tasks. The main idea of decision level fusion method is to fuse the softmax values acquired from different neural networks through mean calculation, or uncertainty reasoning algorithms such as Dempster—Shafer evidence theory (DS theory) and Bayesian Theory [20,21]. The experiment results indicate that merged neural networks with decision level fusion outperform single deep architectures in taxonomic tasks [20,21,22,23].

The main obstacles of current algorithms for ESC tasks are as follows: 1) the most widely used acoustic features applied to ESC tasks were originally designed for ASR and MIR, such as log-mel spectrogram and MFCC. Since the environment sounds are mostly non-stationary signals without meaningful patterns or sub-structures, use a single feature may lead to the failure of capturing important information about environmental audio events. 2) In recent years, with the advancement of deep learning models, the CNN becomes a primary choice in environment sounds recognition and outperform the conventional classifiers like SVM or GMM [7,24]. Even though some research works attempt to use deeper neural networks [25,26] or stacked deep architectures [17,18,19,20] to improve the taxonomic accuracy, however, the performance is still unsatisfactory. Hence, there is need to develop appropriate auditory features and novel neural network models to achieve high categorization accuracy for ESC tasks.

In order to address these two deficiencies, we propose a novel four-layer stacked CNN architecture based on two combined auditory features and DS theory-based information fusion method, called TSCNN. The proposed system consists of three components: feature extraction and combination, CNN training and DS theory-based decision-level fusion. We extract five auditory features: log-mel spectrogram (LM), MFCC, chroma, spectral contrast and tonnetz (in order to facilitate the description in the rest of papers, we call the last features as CST). Then, LM and CST (LMC) are combined as one feature sets, MFCC and CST (MC) are aggregated as another for training two CNNs, respectively. At last, the outputs derived from the softmax layer of these two CNNs are fused by DS theory to exploit both combined features. The experimental results indicate that the taxonomic accuracy of the proposed architecture can surpass both LCNet (CNN use LMC feature) and MCNet (CNN use MC feature), and is also outperforming the existing models on Urbansound8K [27] dataset. To our best knowledge, this is the first time that the classification accuracy of CNN-based ISR system is higher than 97% in ESC tasks.

The remaining structure of this paper is organized as follows. Section 2 introduces the related works on environment sound recognition. Section 3 describes the feature extraction and the architecture of the proposed model. The experiment results and detailed analysis are shown in Section 4. In Section 5, the conclusion of our work is presented.

## 2. Related Works

With the growing number of evidence that the CNN-based models outperform conventional methods in various categorization tasks, they have been applied in sound recognition tasks in recent years. Ref. [28] first evaluated the performance of using CNN in ESC tasks. In this work, an ESC system consists of 2-layer CNN with max-pooling and 2 fully connected layers is proposed. Log-mel spectrograms are extracted as an auditory feature to train the neural network. The experiment results indicate that the classification accuracy of this model is 5.6% higher than traditional methods. Ref. [12] propose to use CNN with smoothed and de-noised spectrogram image feature in sound recognition tasks. Ref. [29] presents a CNN model using mel-spectrograms as features. The performance of three neural network layers as classifiers are investigated, which is a fully connected layer, convolutional layer and convolutional layer without max-pooling. The results indicate that using a convolutional layer as a classifier outperforms the model applying a fully connected layer as the classifier. Ref. [24] presents a six-layer CNN model for acoustic event recognition. In this work, the log-mel spectrograms with their first order derivation and second order derivation are extracted for each recording without segmentation. Then, multiple instance learning is applied and the softmax layer is replaced by an aggregation layer to aggregate the outputs of each network. The data augmentation is applied to prevent over–fitting and improve the robustness of the model. CNN has a strong ability to extract features directly from raw inputs, which has been verified in various image recognition problems. Based on this, Ref. [30] proposes to use CNN to extract features from raw waveform and use SVM or extreme learning machines as classifiers in ESC tasks. The results denote that this architecture outperforms the CNN trained by MFCC. However, the accuracy is only 70.74%. Ref. [26] and uses raw waveforms to train CNNs as well. In this work, the problem of how many layers are the most suitable for CNNs has been studied. With considerable experiments, it is pointed out that deeper layers do not give better performance. Meanwhile, the results also indicate that using waveforms just achieve an approximative performance of models using log-mel features.

Traditional CNN models have several drawbacks for auditory tasks. For example, pooling layers are generally applied in CNN models for feature dimensional reduction, however, these processes can lead to information loss and hinder the performance of neural networks. Therefore, a considerable number of works attempt to use improved CNNs for ESC tasks. Dilated convolution layers are exploited for ESC [31,32] to avoid the above-discussed obstacles. Several research works exploit CNN models which were originally developed for image recognition tasks, and achieve outstanding performance in ESC as well [25]; the environment sound classification accuracy of AlexNet and GoogLeNet [33] are evaluated on UrbanSound8K, ESC-10 and ESC-50 [34] datasets. Spectrograms (Spec), MFCC and Cross Recurrence Plot (CRP) feature sets are extracted and concatenated as three-channel image feature to train both models. The experiment results indicate that the image recognition models could also obtain good taxonomic accuracy for sound recognition problems. The authors in Ref. [35] use an end-to-end ESC system using a convolutional neural network. In this model, raw waveforms are used as inputs and two convolution layers are applied to extract features. Then, three max-pooling layers are performed for feature dimensional reduction followed by two fully connected layers as the classifier. A VGGNet [36] based ESC system is presented by Ref. [6], where the convolution filters are set to 1-D for learning frequency patterns and temporal patterns, respectively. Ref. [37] proposes a CNN based model called WaveNet, which uses multi-scale features to make a CNN that learns comprehensive information of environment sounds. First, features are extracted from one recording through the first convolution layer using three types of filter size. The second convolution layer uses corresponding pooling stride to equal the dimension of these features and then, the three features are concatenated to form the multi-scale features. This feature is further combined with a log-mel spectrogram and perform better than other systems on an ESC-50 dataset. The DS-CNN model presented by Ref. [20] also uses a raw waveform and log-mel spectrogram as inputs to train CNN based ESC system. The difference between WaveNet and DS-CNN is: the WaveNet combined two kinds of features together while in DS-CNN, two different CNN use raw waveform and log-mel spectrogram as inputs, respectively, and the outputs are fused by DS theory.

From these works, we can notice that most ESC models use raw waveform directly or single auditory features to train neural networks. However, after a comprehensive investigation of a considerable number of sound recognition works, Ref. [5] pointed out that aggregate features will give better performance than single features in ESC problems. Meanwhile, from the classification accuracy derived from these recently published works, we can also find out that the CNN-based ESC or ISR systems still has great potentials for making further progress. Hence, we hope to find efficient aggregated features and appropriate CNN architecture to elevate the performance for environment sound categorization.

## 3. Two-Stream CNN with Decision-Level Fusion

In this section, we first describe the feature extraction and combination method. Then, the structure of CNN model and DS theory-based information fusion algorithm will be presented.

### 3.1. Feature Extraction and Combination

Several works [4,9,38,39] have proven that aggregated features achieve higher classification accuracy of environment sounds than single features for both ASR and MIR. The same feature combination methods are introduced in our work to classify environment sounds.

**Figure 1:**
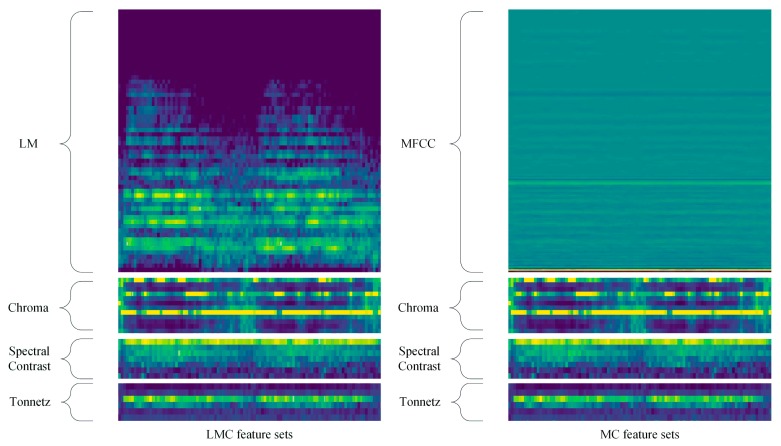
The spectrogram of LMC and MC feature sets.

As log-mel spectrogram and MFCC are the most widely used auditory features in sound recognition, these two feature sets are extracted at first. Then, chroma [40], spectral contrast [41] and tonnetz [42] are extracted through Librosa [43] library. Log-mel spectrogram, chroma, spectral contrast and tonnetz are aggregated to form the LMC feature sets, and MFCC is combined with chroma, spectral contrast and tonnetz to form the MC feature sets. Both feature sets are combined in a linear way, and their time-frequency representations are shown in Figure 1.

### 3.2. Structure of the MCNet and LMCNet

**Figure 2:**
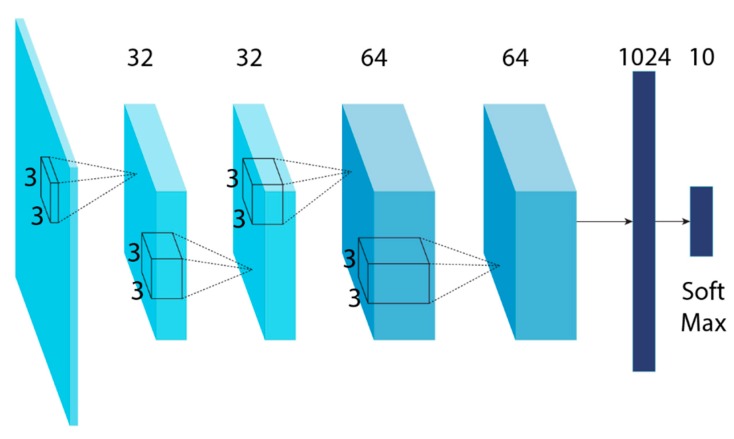
The architecture of proposed four-layer CNN.

The two networks of TSCNN both contain four convolution layers and one fully connected layer. The framework of the proposed four-layer CNN is shown in Figure 2; the architecture of the model is as follows:

(1) The first layer uses 32 kernels with 3×3 receptive field and the stride step is set to 2×2 and batch-normalization is performed. The Rectified Linear Unit (ReLU) is exploited as the activation function.
(2) The second layer uses the same settings as the first layer, where 32 convolution kernels with receptive filed of 3×3 and stride step of 2×2. The batch-normalization is performed and activation function is ReLU as well. The difference is that the second layer applies max-pooling for dimensionality reduction of feature maps.
(3) The third layer uses 64 convolution kernels with a receptive field of 3×3 and the stride step is also 2×2, where batch-normalization is used. Followed by the activation function, ReLU.
(4) The fourth layer 64 convolution kernels with receptive filed of 3×3 and stride step of 2×2. The batch-normalization is performed and activation function is ReLU.
(5) The fifth layer is the fully connected layer with 1024 hidden units and the activation function is Sigmoid.
(6) The output is ten units according to the datasets, followed by the softmax activation function.

At the training stage, we use a 0.5 dropout probability for the second layer, fourth layer and the fully connected layer to prevent overfitting. The CNN is trained through a variant of stochastic gradient descent [44]. The batch size is set to 32, while all weight parameters are subjected to $L_{2}$ regularization and learning rate is set to 0.001 with the momentum of 0.9. The cross-entropy is applied as loss function. At the testing stage, all parameters are the same as the training stage, while the dropout will not be implemented.

### 3.3. Dempster—Shafer Evidence Theory-Based Information Fusion

Dempster-Shafer evidence theory (DS theory) was originally established by Ref. [45], it is also known as belief function theory. The DS theory is mainly about quantified beliefs like Bayesian probability. The main idea of is the notion of evidence and how different pieces of evidence should be combined in order to make inferences [46].

The basis of DS theory is to establish a frame of discernment Θ and a subset of hypothesis ${\{A}_{1}{,\;A}_{2}\ldots A_{n}\}\subseteq \Theta$, where n is the number of hypothesis. $A_{i}$ is an element of the power set P(Θ). Mass function or basic probability assignment M is a mapping: P(Θ)→[0,1] distribute a mass value to each hypothesis $A_{i}\subseteq \Theta$. The mass function represents the trust level of each element itself. There are two constraints of mass function:

(1) $\sum_{A\subseteq \Theta }M(A)=1$, which means the sum of each probability in subset A is 1.
(2) M(∅)=0, this indicate that the mass function cannot allocate any value to an empty set. Meanwhile, a mass function with this characteristic is called normalized mass function.

In this work, the category of sounds in the dataset can be treated as an element in subset A under the frame of discernment Θ. Here, n=10 according to the classes number of UrbanSound 8K and each element are independent. For solving reasoning problems, the mass function representing different part of evidence must be combined in a meaningful way. Here, we use Dempster’s rule to combine the two mass functions derived from each CNN. This combination rule allows combining normalized mass function that are obtained over the same frame of discernment.

The outputs of softmax of LMCNet and MCNet are used as the mass function $M_{1}(B)$ and $M_{2}(C)$. The combination of mass function ($M_{1⊕2}=M_{1}⊕M_{2}$) based on Dempster’s rule ⊕ is defined as:

(1) $$M_{1⊕2}(A)=\alpha \sum_{B\cap C=\emptyset }M_{1}(B_{i})M_{2}(C_{i}),\begin{array}{cc} & \forall A\subseteq \Theta ,A\neq \emptyset \end{array}$$

(2) $$M_{1⊕2}(\emptyset )=0$$

(3) $$\alpha =\frac{1}{\sum_{B\cap C=\emptyset }M_{1}(B_{i})M_{2}(C_{i})}$$

where, α is a normalization constant indicating the mass function is normalized. $M_{1⊕2}(A)$ is a mass function as well and satisfied $\sum_{A\subseteq \Theta }M_{1⊕2}(A)=1$, which is the final probability assignment of $M_{1}(B)$ and $M_{2}(C)$, it is also the result of the fusion process of LMCNet and MCNet.

**Figure 3:**
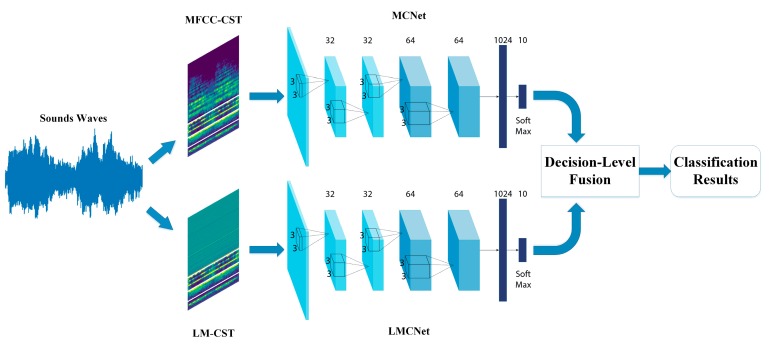
The overall framework of the DS theory based ISR system.

With the LMCNet, MCNet and the DS theory-based information fusion method, we propose the TSCNN. The overall framework of the this ISR system is shown in Figure 3.

From Figure 3, we can see that MFCC, Log-Mel Spectrogram, Chroma, Spectral Contrast and Tonnetz features are extracted from sound waveforms. Then, MFCC and Log-Mel Spectrogram are combined with the rest three features, separately. The MFCC-CST feature set is used to train the MCNet and LM-CST is used to train the LMCNet. Finally, the softmax value derived from each neural network are fused through DS evidence theory to form the sound classification results.

## 4. Experiment and Analysis

The UrbanSound8K dataset includes 8732 labeled urban sounds (the length is less than or equal to 4 s) collected from the real-world, totaling 9.7 h. The dataset is separated into 10 audio event classes: air conditioner (ac), car horn (ch), children playing (cp), dog bark (db), drilling (dr), engine idling (ei), gunshot (gs), jackhammer (jh), siren (si) and street music (sm).

**Figure 4:**
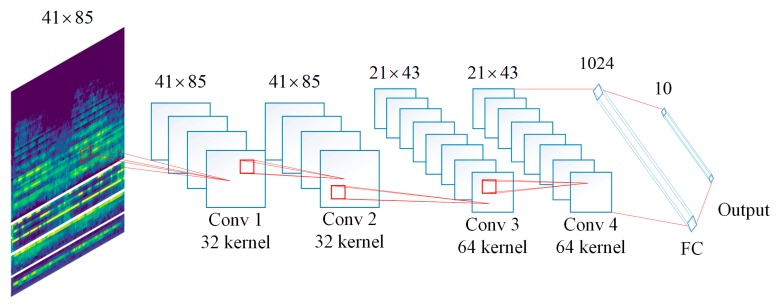
The architecture and size of feature maps in each convolutional layer.

The same feature extraction method presented by Ref. [28] is used in this work. All sound clips are converted to the single channel wave files with the frequency of 22,050 Hz. Then, divided into 41 frames with an overlap of 50% (each frame is about 23 ms). We use the pre-setting channels of Librosa to extract the Chroma, Spectral Contrast and Tonnetz features. For the MFCC extraction, the value of first twenty channels with their first and second order derivatives are used, resulting in 60-dimensional feature vectors. The channels of Log-Mel Spectrogram are set to 60, in order to make the dimension to be equal to the MFCC. Then, all the spectrograms are represented as a matrix with a size of 41×60. The feature size of chroma, tonnetz and spectral contrast is 41×7, 41×6 and 41×12, separately. Therefore, the size of LMC and MC are all 41×85. Figure 4 shows the graphical representation of how does the feature learned by the proposed fourfour-layer CNN.

It can be seen from Figure 4 that the feature maps derived from first and second convolutional layers have the same size as the input feature. After 2×2 max pooling processing, the size of input feature maps for third convolutional layer is 21×43. Since the max pooling is not performed after convolutional layer 3, so that the size of input features for 4th convolutional layer is 21×43 as well. Then, features with size of 11×22 are derived from the last hidden layer and feed to the fully-connected layer which has 1024 hidden units. The output is a 1×10 tensor according to the number of classed of UrbanSound8K dataset is 10.

For each experiment, the ten-fold cross-validation is performed to evaluate the proposed ISR model on UrbanSound8K dataset. The combined features and four-layer CNN architecture are two main contributions of this work. Hence, we first analyze the efficiency of the CNN model trained with combined features. Meanwhile, the influence of the different number of convolution layers (six and eight) on CNN-based ESC system is also investigated. The additional convolution layers in the CNNs for comparison use the same receptive fields of 3×3 and stride step of 2×2, batch-normalization is performed on each layer with ReLU as activation function. Dropout with a rate of 0.5 is exploited for the sixth and eighth convolution layer in the two additional CNN models, respectively. Table 1 presents the number of parameters and the memory cost of CNN with different number of convolutional layers.

**Figure 5:**
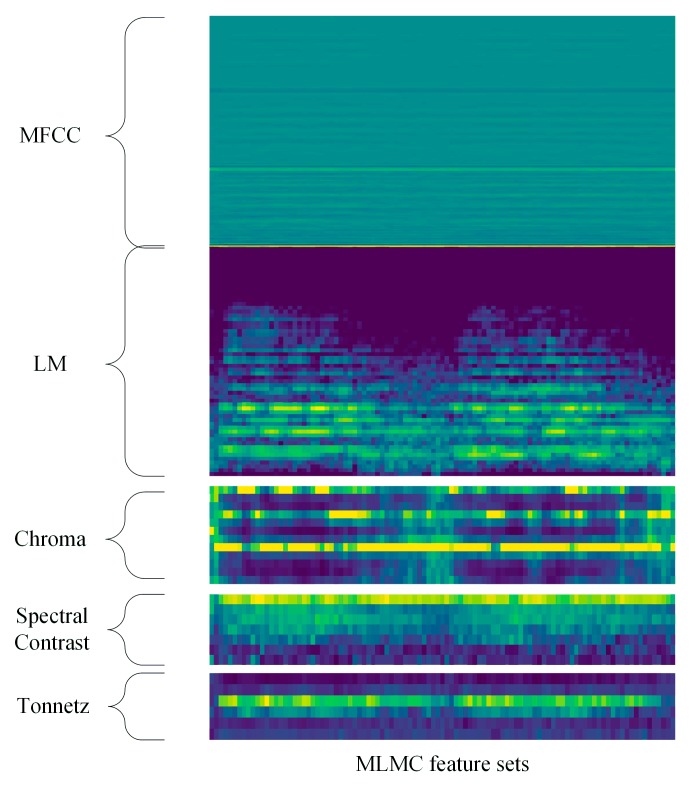
The spectrogram of MLMC feature sets.

Furthermore, the classification performance of feature level fusion method is also presented. We combined LM, MFCC and CST together to form a new feature set called MLMC, to make a further investigation of the influence of various feature combination strategies in ESC tasks. The feature size of MLMC is 41×145. The spectrogram of MLMC is shown in Figure 5. The experiment results are shown in Tables 2, 4 and 5. The class-wise classification accuracy and the average accuracy of ten-fold cross-validation of three combined features and the proposed TSCNN-DS model on UrbanSound8K dataset is presented in each table.

The Table 2 describes the experimental results of each method with four-layer CNN models. We can find that the feature combination of LMC and MC performs well in the four-layer CNN based ISR system. Taxonomic accuracy of five and six classes are higher than 95% using LMC and MC, respectively. While the feature aggregated of all feature sets not only reduces the performance but also makes it slightly worse. The LMCNet and MCNet achieves 95.2% and 95.3%, which is 22.5% and 22.6% higher than the model presented in Ref. [28], respectively. The feature combination of MLMC has the worst performance among the four models, however, it is still 21.9% higher than the 72.7% of Piczak’s model. It can be seen that for both methods, the classification accuracy of all categories is higher than 90% except for gunshot of LMC and MLMC. The proposed TSCNN-DS model reaches 97.2% which is 24.5% higher than Piczak’s work, and it significantly improved the classification accuracy of gunshots (95.4%).

Moreover, in order to further illustrate whether the proposed TSCNN-DS model outperform LMCNet, MCNet and four-layer CNN using MLMC feature sets, we show the standard deviation and time cost in Table 3. The classification accuracy obtained by TSCNN-DS is 2% and 1.9% higher than LMCNet and MCNet. It is also shown in Table 3 that the standard deviation of TSCNN-DS is much less than three other methods, which further demonstrate that the fusion model outperforms three other single models. The mean time cost for LMCNet, MCNet, MLMC and TSCNN-DS is 0.023 s, 0.024 s, 0.028 s and 0.077 s, separately. The test is down in Python under Microsoft Windows 10 x64 OS on a computer with Intel Core i7-8700 CPU, two GTX 1080 GPU (the memory of each GPU is 8 GB) and 32 GB RAM. Although the time cost of proposed model is almost three times longer than single neural networks, the computational cost of TSCNN-DS is still well acceptable for ESC tasks in real time.

It can be seen in Table 4 that, the six-layer CNN based models performs slightly worse than the methods use four-layer CNN. The LMCNet, MCNet, MLMC-CNN and TSCNN is 2.2%, 6.0%, 1.9% and 2.3% worse when compared with the four-layer CNN-based models. The categorization accuracy of gunshot for both methods is less than 90% and it is less than 80% for LMC and MC feature sets. Classification accuracy of dog barking with MCNet failed to reach 90%, and taxonomic accuracy on children playing of MCNet dramatically reduced to 69.4%. The MLMC feature cannot improve the classification performance as well, where the accuracy of children playing and gunshot failed to reach 90%. The same situation also appeared in the TSCNN model. Nevertheless, the proposed TSCNN model still achieves the best classification result (94.9%).

From Table 5 we can find that the performance of all methods is unsatisfactory with the eight-layer CNN. Most of the categories and all methods obtain a taxonomic result that less than 90%. This indicates that using deeper layers may not give a better result for deep architectures, while appropriate layers and suitable parameter settings are the most important components of deep learning models.

In general, we can find out that the proposed LMC and MC features present to be efficiency with the proposed ISR system, which clarifies the advantage of the proposed feature combination strategies in ESC tasks. The TSCNN-DS model outperforming other models for both CNN architectures with different convolution layers. Then, the four-layer CNN achieves the best taxonomic accuracy when compared with six-layer and eight-layer CNN models for both methods. Meanwhile, the classification accuracy of both methods with the proposed four-layer CNN are higher than existing models. These results demonstrate the efficiency of the proposed four-layer CNN and DS theory fusion method based TSCNN-DS model.

In order to make a comprehensively comparison, we also investigate the two-stream CNN with the layer stack method. This model combined the outputs of the second convolution layer of both CNN and the concatenate feature maps are than used as inputs for the next convolution layers. We test this stacked CNN with 4, 6 and 8 layers as well. The parameter settings of each convolution layers and fully connected layers are equal to the 4-, 6- and 8-layer CNN described above. The classification accuracy of these stacked CNNs on UrbanSound8K dataset are shown in Table 6.

It is clearly that the stacked four-layer CNN models achieve the highest (86.4%) classification accuracy among the three models. Which is 6.6% and 6.3% higher than stacked six- and eight-layer CNN respectively. This result further proves that the proper number of layers and parameters is the key to the deep learning model based ISR system, where the advantage of the proposed four-layer CNN is further proved as well.

At last, we compare our TSCNN-DS model with several existing CNN based ISR models as presented by Refs. [6,20,25,28,32,35]. The results are shown in Table 7. The LMCNet uses LMC feature sets and achieves an accuracy of 95.2%, which is 22.5% higher than the Ref. [28] model that uses LM features. Meanwhile, it is 11.5% higher than the Ref. [32] model and uses LM and Gammatone Spectrogram combined feature. Furthermore, the performance of LMCNet is slightly higher (3%) than the model presented by Ref. [20], which also applies DS theory as a sfusion method to fuse two CNN models. The classification accuracy of MCNet is 95.3%, which is much higher than the 72.7% of the model proposed by Ref. [28]. Moreover, the proposed MCNet is also significantly higher than the Ref. [28] model and is 2.3% higher than the Ref. [25] model with MFCC based aggregated featurs. Finally, the proposed DS theory-based TSCNN-DS model obtains the highest taxonomic accuracy (97.2%) among all the ESC models. The performance of our algorithm is much higher than the Ref. [28] model and is 5% higher than the Ref. [20] model which uses the same fusion strategy. To our best knowledge, this is the first time that the categorization accuracy has reached over 95% on the UrbanSound8K dataset and is the highest accuracy compared with existing models.

## 5. Conclusions

In this paper, we proposed the TSCNN-DS model of intelligent sound recognition problems. It consists of two four-layer convolutional neural networks, the LMCNet and MCNet trained by two combined features, LMC and MC feature sets, respectively. Then, the outputs of the softmax layer of both networks are fused through DS evidence theory; the result is the predicted categorization of an environment sound. The performances of two CNNs with the novel combined feature sets and the entire framework are tested on UrbanSound8K dataset and compared with existing models which published in recent years. The LMCNet and MCNet reaches 95.2% and 95.3% on UrbanSound8K dataset, which is 22.5% and 22.6% higher than the Piczak’s model [28], respectively. Meanwhile, these two neural networks are all slightly higher than recent ESC models which use same feature (LM or MFCC) to form a combined eigenvector. These results indicate that the proposed CNN is more effective for environment sounds classification tasks according to the appropriate parameter settings and a comprehensive representation of sound recordings through the combined feature sets. The TSCNN-DS achieves 97.2% on the UrbanSound8K dataset, which is 4.2% higher than the state-of-art methods (the Ref. [25] model), and is 5% higher than the Ref. [20] model where the same fusion algorithm is exploited to fuse two CNNs.

The DS theory can substantially improve the taxonomic performance of the single CNN model in ESC problems, however, from Table 2 we can find out that the accuracy of repeated discrete sounds (car horn, dog barging and gunshot) is worse than other sound classes. This is maybe caused by the number of convolutional layers, which prevents the model from extracting enough feature maps to comprehensively represent important information of sound signals. Another probability is the feature (LC and MC) which may neglect some needed information for representing such discrete sound signals. To improve the categorization accuracy on these kinds of sounds with the TSCNN-DS model will be among our future work. Both new feature extraction methods and novel CNN architectures should be established for conquering these problems and improving the classification performance. Meanwhile, the computation cost should also be considered to make an ISR model which can be applied in real time.

## Figures and Tables

**Table 1:** Parameters and memories of CNN with different number of convolutional layers.

	four-layer		6-Layer		8-Layer	
Layer	param	memory	param	memory	param	memory
input	0	3.5 K	0	3.5 K	0	3.5 K
Conv 3×3−32	288	111.5 K	288	111.5 K	288	111.5 K
Conv 3×3−32	9.2 K	111.5 K	9.2 K	111.5 K	9.2 K	111.5 K
Conv 3×3−64	18.4 K	57.8 K	18.4 K	57.8 K	18.4 K	57.8 K
Conv 3×3−64	36.8 K	57.8 K	36.8 K	57.8 K	36.8 K	57.8 K
Conv 3×3−128	0	0	73.7 K	31 K	73.7 K	31 K
Conv 3×3−128	0	0	147.5 K	31 K	147.5 K	31 K
Conv 3×3−256	0	0	0	0	294.9 K	4.6 K
Conv 3×3−256	0	0	0	0	589.8 K	4.6 K
Fc 1024	15.9 M	1024	8.7 M	1024	4.7 M	1024
Fc 10	10.2 K	10	10.2 K	10	10.2 K	10
Total	15.9 M	339.6 K	8.9 M	401.6 K	5.9 M	413.4 K

**Table 2:** Class-wise accuracy of four models with four-layer CNN evaluated on UrbanSound8K.

Class	LMC (LMCNet)	MC (MCNet)	MLMC	TSCNN-DS
ac	98.6%	99.9%	99.2%	99.9%
ch	93.9%	91.4%	93.2%	94.2%
cp	97.3%	93.9%	96.1%	97.5%
db	92.6%	90.4%	94.2%	95.3%
dr	94.8%	95.0%	95.7%	97.2%
ei	98.9%	99.6%	98.5%	99.6%
gs	88.6%	91.1%	85.9%	95.4%
jh	93.2%	95.9%	91.1%	97.1%
si	98.6%	98.3%	98.5%	98.9%
sm	95.0%	97.4%	94.1%	96.9%
Avg.	95.2%	95.3%	94.6%	97.2%

**Table 3:** Statistics analyze and time cost of four-layer CNN based models and TSCNN-DS model.

	Mean	N	Std Deviation	Time Cost
LMCNet	0.9515	10	0.03121	0.023
MCNet	0.9529	10	0.03352	0.024
MLMC	0.9465	10	0.03812	0.028
TSCNN-DS	0.9720	10	0.01788	0.077

**Table 4:** Class-wise accuracy of four models based on 6-layer CNN evaluated on UrbanSound8K.

Class	LMC (LMCNet)	MC (MCNet)	MLMC	TSCNN-DS
ac	98.9%	98.9%	97.5%	99.9%
ch	90.2%	69.4%	87.9%	89.2%
cp	94.8%	91.1%	93.6%	96.4%
db	91.3%	88.0%	91.6%	93.1%
dr	93.8%	90.9%	91.5%	95.5%
ei	98.2%	97.7%	98.1%	99.1%
gs	77.2%	77.2%	81.7%	85.1%
jh	92.6%	91.6%	93.4%	97.1%
si	99.0%	96.1%	99.0%	98.9%
sm	94.3%	92.1%	92.9%	94.7%
Avg.	93.0%	89.3%	92.7%	94.9%

**Table 5:** Class-wise accuracy of four models based on 8-layer CNN evaluated on UrbanSound8K.

Class	LMC (LMCNet)	MC (MCNet)	MLMC	TSCNN-DS
ac	94.8%	91.5%	93.2%	98.2%
ch	76.1%	47.3%	88.1%	69.9%
cp	84.0%	80.9%	87.9%	88.0%
db	79.9%	73.3%	86.8%	80.8%
dr	87.8%	87.4%	87.0%	91.6%
ei	96.8%	94.8%	95.3%	97.4%
gs	57.2%	63.4%	45.4%	67.8%
jh	89.8%	74.7%	85.9%	87.6%
si	97.8%	88.3%	96.5%	96.3%
sm	85.3%	71.8%	90.3%	80.3%
Avg.	84.9%	77.3%	85.7 %	85.8%

**Table 6:** The ESC results of stacked CNNs with 4, 6 and 8 convolution layers.

Model	Accuracy
Stacked four-layer CNN	86.4%
Stacked 6-layer CNN	79.8%
Stacked 8-layer CNN	80.1%

**Table 7:** Comparison of classification accuracy with other models on UrbanSound8K datasets. The bold is our result.

Model	Feature	Accuracy
Piczak [28]	LM	72.7%
Tokozume [35]	Raw Data	78.3%
Zhang X. [32]	Mel	81.9%
Zhang Z. [6]	LM-GS	83.7%
Li [20].	Raw Data-LM	92.2%
Boddapati [25]	Spec -MFCC-CRP	93%
LMCNet	LM-C	95.2%
MCNet	M-C	95.3%
**TSCNN-DS**	**MC and LMC**	**97.2%**
